# Inferring Gene Regulatory Networks by Singular Value Decomposition and Gravitation Field Algorithm

**DOI:** 10.1371/journal.pone.0051141

**Published:** 2012-12-04

**Authors:** Ming Zheng, Jia-nan Wu, Yan-xin Huang, Gui-xia Liu, You Zhou, Chun-guang Zhou

**Affiliations:** 1 College of Computer Science and Technology, Jilin University, Changchun, People's Republic of China; 2 College of Computer Science and Technology, Changchun University, Changchun, People's Republic of China; 3 National Engineering Laboratory for Druggable Gene and Protein Screening, Northeast Normal University, Changchun, China; National Institute of Environmental and Health Sciences, United States of America

## Abstract

Reconstruction of gene regulatory networks (GRNs) is of utmost interest and has become a challenge computational problem in system biology. However, every existing inference algorithm from gene expression profiles has its own advantages and disadvantages. In particular, the effectiveness and efficiency of every previous algorithm is not high enough. In this work, we proposed a novel inference algorithm from gene expression data based on differential equation model. In this algorithm, two methods were included for inferring GRNs. Before reconstructing GRNs, singular value decomposition method was used to decompose gene expression data, determine the algorithm solution space, and get all candidate solutions of GRNs. In these generated family of candidate solutions, gravitation field algorithm was modified to infer GRNs, used to optimize the criteria of differential equation model, and search the best network structure result. The proposed algorithm is validated on both the simulated scale-free network and real benchmark gene regulatory network in networks database. Both the Bayesian method and the traditional differential equation model were also used to infer GRNs, and the results were used to compare with the proposed algorithm in our work. And genetic algorithm and simulated annealing were also used to evaluate gravitation field algorithm. The cross-validation results confirmed the effectiveness of our algorithm, which outperforms significantly other previous algorithms.

## Introduction

With recent advances in large-scale gene sequencing technology, especially mRNA [1] hybrid microarray, it has become possible to study life phenomenon and essence in genome-scale data. Much data can be used to calculate various complex problems. And its possible to infer gene regulatory networks (GRNs) [2] from various large-scale gene expression data. And the study of reconstruction algorithms from large-scale gene expression profiles has become the research focus point in system biology. To extract the topology of GRNs and gene regulation relationships in GRNs is a computationally daunting task.

Recently, many current research efforts have focused on various models of inferring GRNs from genome-wide scale gene expression data. These inference models include Boolean network model [3], Bayesian network model [4], neural network model [5], Differential equation model (DEM) [6], and so on. In these models, the most accuracy model is DEM. The data used for DEM can be downloaded from GEO database [7].

But DEM typically requires a large amount of data to compute the connected network, even the genomic-scales data. To overcome the data shortage problem and computational inefficiency of DEM, two methods must be used in DEM procedure. One is singular value decomposition (SVD) method [8] used to construct a family of candidate solutions. The other is a novel heuristic search algorithm gravitation field algorithm used to find the optimal GRN structure.

SVD had been adopted to infer GRNs in some research, but traditional SVD method can only provide one solution for GRN, which may be not the best one in solution space. An improved SVD should be used to get all legal candidate solutions, and eliminate all illegal solutions. Other extra methods have to be added in the SVD procedure, such as DEM and some heuristic search algorithms to infer GRNs. The number of legal solutions for SVD and DEM may be infinite. But the number of best solutions may be only one or several. A suitable heuristic search algorithm should be used for searching the best solutions. Genetic algorithm (GA) [9], simulated annealing (SA) [10], particle swarm optimization (PSO) [11] and other algorithms do not comply with the reconstruction of GRNs algorithms. Because the connectivity of GRNs is equal to squared nodes, a novel algorithm called gravitation field algorithm (GFA) [12], which can resolve large-scale computational problems, should be used in our work.

**Figure 1:**
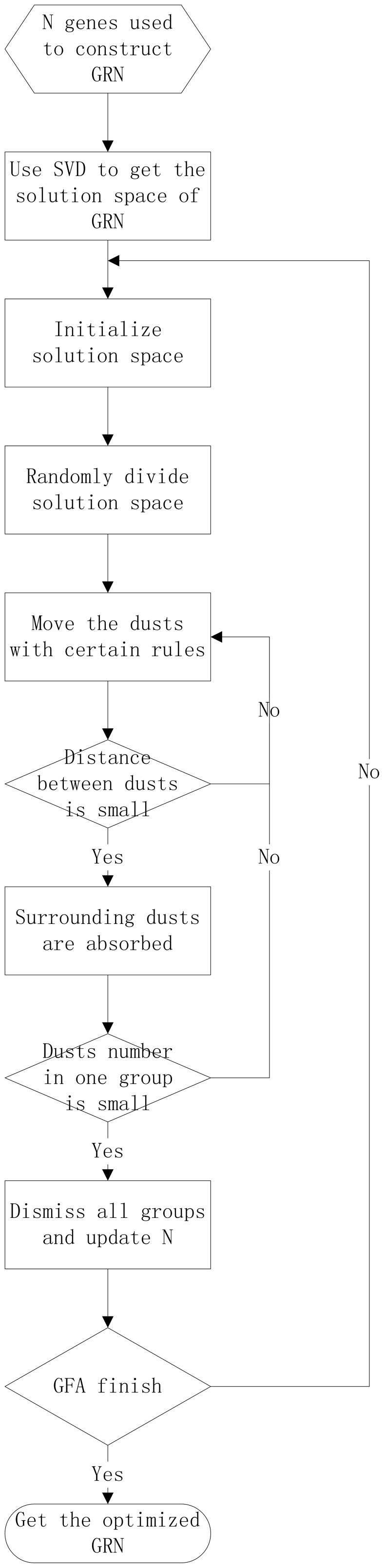
Flow chart of the algorithm.

Characterized by stability, fast operation and efficiency for simple object functions, GFA is suitable for inferring GRNs. But GFA can only be used to analyze continuous data, two parts in GFA will be modified to resolve discrete data problem. The first part was the dusts initialization in discrete solution space. The other one is the movement operator which includes four steps to move the dusts and search optimal solutions.

**Figure 2:**
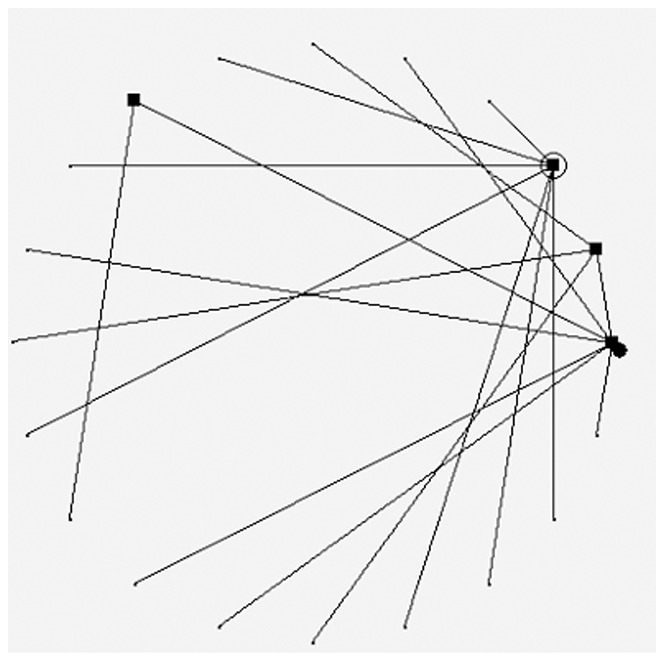
A typical scale-free figure generated by reconnected algorithm.

The proposed algorithm is validated on both the simulated scale-free network and real benchmark gene regulatory network. The simulated gene expression data were generated from scale-free network. The real gene expression data were downloaded from online database. 500 different runs were used in this paper. And the results were compared with GA, SA, Bayesian model and traditional DEM. The cross-validation results confirmed the effectiveness of our algorithm, which outperforms significantly other previous methods. Besides its high accuracy, the running-time of our algorithm is also significantly quick.

**Table 1:** part of gene expression data of generated GRN.

Nodes	Time0	Time0	Time0	Time0
**Node0**	0.329	0.135	0.358	0.251
**Node1**	0.934	0.439	0.314	0.900
**Node2**	0.911	0.708	0.627	0.600
**Node3**	0.501	0.595	0.530	0.527

## Methods

### Solution space determination with SVD

DEM can be defined as the gene expression change rate. The gene expression derivative of any gene i at time t is shown as Eq. (1):
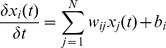
(1)


In Eq. (1), 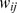 is the weight value of influence from gene j to gene i. All N 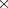 N weight values were the elements of the weight matrix W. N is the number of nodes in the GRN. GRNs are networks with directivity, so 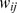 is not equal to 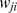. The meanings of these two weight values are opposite. 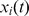 represents the expression of gene i at time t. 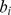 is the expression of gene i, which is the expression of gene i with no external interference.

**Figure 3:**
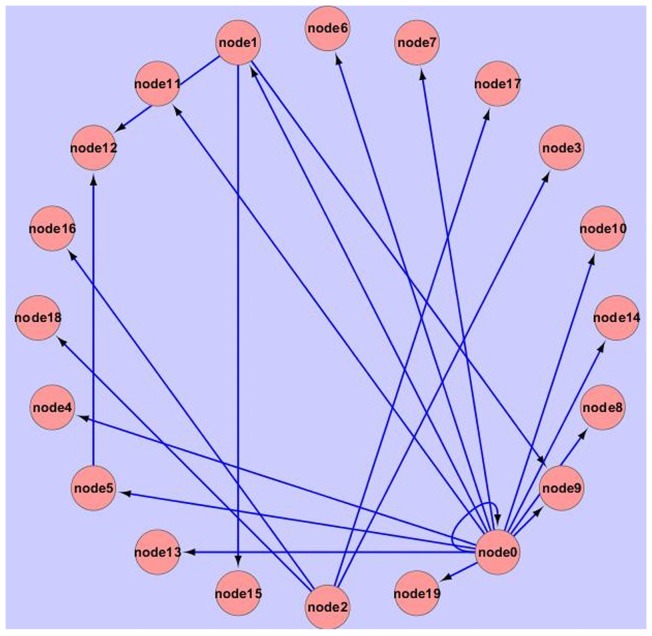
GRN of simulated data based on our algorithm.

The gene expressions of N genes and T times can be defined as a matrix Eq. (2):
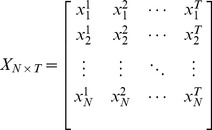
(2)


In Eq. (2), 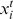 represents the expression of gene i at time t. According to Eq. (2), Eq. (1) can be deduced as Eq. (3):
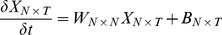
(3)


The goal of every inference algorithm is to use the measured data X, X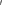 and B to deduce the connectivity weight value matrix W. SVD method is used to decompose X into Eq. (4):
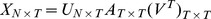
(4)


In Eq. (4), both V and U are orthogonal matrices, and V and U comply with Eq. (5):
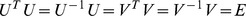
(5)


In Eq. (5), E is an identity matrix, and the transpose matrices of V and U are equal to the inverse matrices of V and U. Eq. (5) must be in the generalized inverse matrix definition [13]. And matrix W can be calculated from Eq. (3) as Eq. (6):
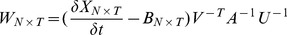
(6)


Eq. (6) is one solution of GRN which is generated by SVD method from gene expression data X. But this solution may be not the best one in DEM. Further calculation in SVD method should be done to get all legal solutions in linear algebra rules. Eq. (7) calculated from Eq. (6) contains all solutions:
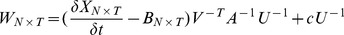
(7)


In Eq. (7), c is an arbitrary value. Eq. (7) is a general solution of DEM with the special solution Eq. (6). In mathematics, Eq. (7) reduces the solution space of DEM and generated all candidate solutions. And optimization operation can be done in these candidate solutions.

**Table 2:** The compared results table of reconstruction of GRN with three algorithms.

algorithms	PPV	Se	running time
**GFA**	0.329	0.735	2.58
**GA**	0.304	0.639	4.35
**SA**	0.286	0.595	12.90
**Bayesian**	0.237	0.489	3.57
**DEM**	0.316	0.617	4.69

### GRN optimization with improved gravitation field algorithm

After all candidate solutions of DEM are calculated with SVD, optimization within the value domain will begin. The criterion energy function used to select the best GRN result in DEM is a key problem. GFA was used to optimize the energy function in our paper. GFA is derived from the point of the hypothesis theory Solar Nebular Disk Model (SNDM) [14]. The algorithm goal is to search the optimal solution of given function or problem. To start with, all the solutions, which are the dusts in the algorithm model, are initialized randomly, or based on the prior knowledge. Whats more, we assign every dust (solution) a weight, we call it mass, whose values are based on the mass function generated from the criteria function. Finally, the power of the dust attraction, which belongs to a certain dust and exists between every two dusts, pulls other dusts to the dust. Hence, the dusts assemble together, and the planets come out in the endthey are the optima. The mathematical proof demonstrates that GFA could be convergent in the global optimum by probability 1 in three conditions for one independent variable mass functions [12]. Least squared function shown as Eq. (8) is used as mass function in our work:

**Figure 4:**
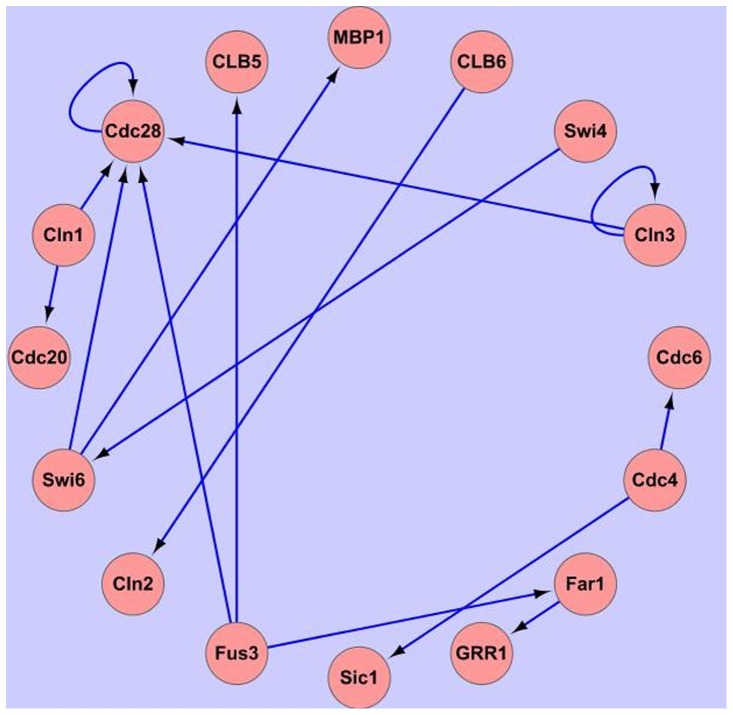
GRN of a part of GDS38 data based on our algorithm.



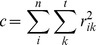
(8)In Eq. (8), c is the difference between the experiment observed value and the algorithm calculated value. 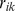 is the residues of DEM. And it is defined as Eq. (9):
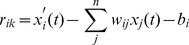
(9)


In Eq. (9), 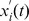 represents the gene expression derivative of gene i at time t, which is shown as Eq. (10):
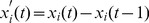
(10)


To search the minimum of Eq. (8), random weight value matrices were used as dusts in GFA. The dimensionality of dusts is the same as W. But the mass function value of one matrix is still a scale value since Eq. (8) is an accumulation function. And this value is used as the criterion of one dust is good or bad. The value domain of each dimension is unconstrained. Actually, the absolute value of each dimensional value will be less than 100 in most cases. Every dimensionality is constrained in [−100, 100] in our work. The algorithm flow is described as following:

**Table 3:** The compared results table of reconstruction of GDS38 GRN with three algorithms.

algorithms	PPV	Se	running time
**GFA**	0.286	0.658	2.36
**GA**	0.238	0.628	4.28
**SA**	0.196	0.576	11.20
**Bayesian**	0.229	0.483	3.57
**DEM**	0.307	0.594	4.69

1. In 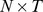 epochs, every element value of one dust is assigned by a random value in [−100, 100].
2. After 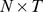 elements of one dust are randomly initialized, the dust will be as the parameter of Eq. (7). If the constant c can be calculated, the initialization will be completed. Otherwise, the algorithm will go to (1), and the dust will be initialized again.
3. Repeat (1) and (2), until all dusts are initialized.

After three parts described above are calculated in GFA initialization step, SVD results can be used as the solution space in GFA. The running time of GFA will be reduced, and the operating efficiency will be also improved.

The random strategy was used in the division part, because the form of dusts was more complex, and the average strategy [12] was not suitable in the GRN inference algorithm. After division, the centre dust which has the optimal (minimum or maximum) mass value in its own group was selected. The movement operation is special, and the procedure is described above:

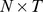
 corresponding elements-pairs between the centre dust and the selected surrounding dust are compared. If the surrounding dust elements are not equal to the centre dust elements, the element values of the surrounding dust will move to the element values of the centre dust. Many methods can be used as movement strategies, such as constant length movement and variable length movement [12].
2. After 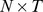 comparisons and movements, the new dust will be as the parameter of Eq. (7) to verify the new dust is legal or not. If the constant c can be calculated, the algorithm goes to (4), otherwise the algorithm goes to (3).
3. The movement pace will be decreased to a half or one third of original pace value. If P (P is a constant number) times movements later, the suitable movement pace is still not found in the algorithm, the surrounding dust will be deleted and absorbed.
4. If the mass value of the new dust is optimized better than the mass value of the centre dust. The new dust will replace the original centre dust, and the original centre dust will be one surrounding dust. And a new movement procedure will begin again between the centre dust and all surrounding dusts in one group.

The method that the surrounding dusts will be deleted directly can be used as the absorption strategy. In the weight value matrix, negative values represent inhibition which is negative regulation. And positive values represent activation which is positive regulation.

In the procedure of GRN reconstruction, the algorithm task is to find a suitable fully connected network. And the self-regulation is also considered in the algorithm. Actually, GRN is not a fully connected network structure, but a sparse structure. There are many methods to resolve this problem. The easiest but efficiency method is threshold interception method. A threshold, such as 0.5, is used to determine the influence link is existed or not. If the absolute value of the weight value is less than 0.5, the link will be deleted. Otherwise the link is existed.

We use SVD to reduce the solution space, GFA to optimize the network structure, DE to deduce the influence relationship in each genes-pair. The whole flow chart of the proposed algorithm is shown as Fig. 1.

## Results

The proposed algorithm is validated on both a simulated scale-free network and a real benchmark gene regulatory network. Both genetic algorithm and simulated annealing were also used to calculate and compare with the proposed algorithm.

### Simulated data experiment

First of all, simulated data were used to verify the proposed inference algorithm. The simulated data were generated with the reconnection algorithm [6]. The gene regulatory scale-free network [15] were generated firstly. The regulatory node of the new added node will be reselected according to the reconnection method. The probability of reconnection depends on power-law distribution model parameters. Then reverse DEM method was used to get the corresponding gene expression data. In the simulated scale-free network, the amount of control nodes is limited. The links of any node will be in the power-law distribution. A typical generated scale-free network is shown as Fig. 2.

In Fig. 2, rectangle nodes represent GRN regulatory nodes, empty circle nodes represent self-regulatory nodes, the only one filled circle node represents node 0. And the nodes counterclockwise from node 0 are node 1, 2, until node 19. The corresponding gene expression data were generated, and parts of the data were shown as Table 1.

When generating data, a random perturbation value generated from uniform distribution was added to the DE solutions to make special different expression value. The gene expression data like Table 1 were analyzed by the algorithm proposed in this paper, and used for inferring GRN. Finally, weight value matrix W was generated and the GRN was shown through free software Cytoscape [16] as Fig. 3. In Fig. 3, arrow lines represented the influence relationship. In Cytoscape, the Circle topology algorithm [17] was used to layout these nodes, so the nodes sequence were changed, but the accuracy and the running time of the algorithm would be not changed.

In order to validate our algorithm and compare the efficiency of GFA in GRN reconstruction algorithm, positive predictive value (PPV) and Sensitivity (Se) function were used as the criteria defined by Eq. (11) and Eq. (12):
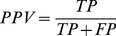
(11)

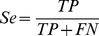
(12)


In Eq. (11) and Eq. (12), TP =  True Positive, which represents that the calculated results were true, and the real experimental results were also true. FP =  False Positive, which represents that the calculated results were false, but the real experimental results were true. FN =  False Negative, which represents that the calculated results were true, but the real experimental results were false. From these two function, we can see that, PPV and Se is bigger, the efficiency of the algorithm is higher. And running time of the algorithm is also used as criterion for efficiency. One our algorithm result was shown as Fig. 3.

GFA, GA and SA were used in the GRN reconstruction algorithm. All these three optimal methods were based on both DEM and SVD, and the optimal algorithms were used to search the best GRN. Besides this comparison, Bayesian network and tradition DEM were also used to test our algorithm. All GRN results were calculated and summarized. 500 different runs were measured, and different results were average and shown as Table 2.

In Table 3, the unit of running time is second. From this table, we can conclude that PPV and Se of GFA had more some advantages than other two optimal algorithms. And the running time is shorter than GA and SA. That is, the efficiency of GFA is higher. GA is good also. SA is the worst; especial the running time of SA is too long.

The running-time of Bayesian algorithm is less than the traditional DEM, but PPV and Se of Bayesian is less than the traditional DEM. Our algorithm, which is GFA, is better than both Bayesian and traditional DEM in PPV, Se and running-time criteria.

### Real data experiment

Besides the simulated data experiment, real data experiment was also used to identify how well it works in constructing regulatory networks. In this part, the well-known yeast (Saccharomyces cerevisiae) cell cycle microarray time series dataset [18] were used. The data can be downloaded from GEO database [7], and the series number is GDS38. GDS38 consists of three sub-sets measured using different cells synchronization methods. As others did, only a part of the yeast cell cycle pathway was selected in KEGG to test the proposed algorithm. The corresponding GRN figure can be seen from KEGG database [19]. This selected sub-network contains 16 genes, and the result was shown with Cytoscape as Fig. 4.

Fig. 4 is the GRN reconstruction result of 16 genes calculated by GFA. To get the statistical results, 500 different runs with GFA, GA, SA, Bayesian and traditional DEM were measured and summarized. Assuming that this GRN in KEGG reflects biological reality, we can count the number of TP, FP and FN and calculate PPV and Se as it is done for artificial systems. The results were shown as Table 3.

From Table 3, we conclude that the proposed algorithm outperforms Bayesian and traditional DEM. And GFA is better than both GA and SA. But accuracy of the real data is lower than the simulated data, because the interference of simulated data is less than the real data. But the compared results were also proved that the proposed algorithm is better than others in PPV and Se criteria.

In one word, GFA will be a key in the GRN reconstruction algorithm. The running time is less, and the accuracy is higher.

## Discussion

To improve the accuracy and reduce the running-time of GRN reconstruction algorithm, a novel algorithm for inferring GRNs from gene expression data was proposed and used in this work. In this algorithm, two methods were taken into account in the procedure. Before reconstructing operation, singular value decomposition method was used to construct the algorithm solution space. In the generated family of candidate solutions, gravitation field algorithm was modified for inferring gene regulatory network, and used to optimize the criteria of differential equation model and find a best network structure result.

In the experiments, both the cross-validation results and comparison results for reconstruction of GRN demonstrate the effectiveness and efficiency of our algorithm. Besides its high accuracy, the running-time of our algorithm is also significantly quick.
